# Effects of Smoking and Age on Chronic Obstructive Pulmonary Disease in Japan

**DOI:** 10.2188/jea.15.113

**Published:** 2005-08-30

**Authors:** Shigeko Kojima, Hiroki Sakakibara, Shinichi Motani, Kunihiko Hirose, Fumio Mizuno, Madoka Ito, Shuji Hashimoto

**Affiliations:** 1 Division of Hygiene, Department of Medicine, Fujita Health University.; 2 Division of Respiratory Medicine and Clinical Allergy, Department of Internal Medicine, Fujita Health University.; 3 Toyota Community Medical Center.

**Keywords:** The Global Initiative for Chronic Obstructive Lung Disease, Pulmonary Disease, Chronic Obstructive, Smoking, Age Factors

## Abstract

BACKGROUND: The Global Initiative for Chronic Obstructive Lung Disease guidelines recommended a forced expiratory volume at one second per forced vital capacity as a standard diagnostic criterion of chronic obstructive pulmonary disease (COPD). A few reports on the risk factors of COPD have used the standard diagnostic criteria. In our study, the effects of age and smoking on COPD in Japan under the standard diagnosis criteria were evaluated.

METHODS: Subjects were 11,460 participants aged 25-74 years during health check-ups including spirometry at the Toyota Community Medical Center in Japan. Logistic regression analyses with or without COPD as a dependent variable and age as an independent variable were conducted among non-smokers. The ratio of the observed number of COPD cases in former and current smokers to the number expected for non-smokers with the same distribution of age (O/E) was calculated.

RESULTS: The proportion of males incurring COPD significantly increased with age, and the O/E for former and current male smokers was significantly higher than one, i.e., O/E (95% confidence interval) for current smokers with a Brinkman Index of <400, 400-799, and 800+ were 3.10 (2.00-4.81), 2.78 (2.05-3.73), and 4.76 (3.65-6.19), respectively. Among females, the O/E for current smokers with a Brinkman Index of <400, and 400-799 were significantly higher than one.

CONCLUSION: Age and smoking were shown to constitute strong risk factors for COPD under the standard diagnostic criteria.

The Global Initiative for Chronic Obstructive Lung Disease (GOLD) guidelines, jointly sponsored by the U.S. National Heart, Lung, and Blood Institute and the World Health Organization, was released in 2000. Although there have been several discussions regarding the diagnosis of chronic obstructive pulmonary disease (COPD),^1^^-^^3^ the GOLD guidelines recommended a forced expiratory volume at one second per forced vital capacity (FEV_1_/FVC) < 70% as follows: “For the diagnosis and assessment of COPD, spirometry is the gold standard as it is the most reproducible, standardized, and objective way of measuring airflow limitation. The diagnosis is confirmed by an objective measure of airflow limitation, preferably spirometry. An FEV_1_/FVC <70% to defining an early sign of airflow limitation is a pragmatic one in view of the fact that universally applicable references values for FEV_1_ and FVC are not available.”^4^ Several epidemiologic and clinical studies have reported that the major risk factors for COPD were smoking and age.^5^^-^^15^ However, the standard criterion of the GOLD guidelines was used in a few studies in Europe and the United States.^12^^,^^15^

In the present study, the standard criterion on the GOLD guidelines for the diagnosis of COPD is used, and the effects of age and smoking on COPD in Japan are evaluated.

## METHODS

### Subjects

Subjects in this study were participants aged 25-74 years subjected to health check-ups including spirometry at the Toyota Community Medical Center in Japan from April 2001 through March 2002. Out of 11,839 participants, 149 with asthma and 240 with tuberculosis were excluded because of the difficulty involved in diagnosing COPD by spirometry; this left 11,460 participants for analysis. Table 1 shows the number of subjects grouped by sex and age at 5- year intervals. The number of subjects thus grouped ranged from 31 to 1,611.

**Table 1.** Number of subjects.

Age (years)	Males	Females
Total	7,574	3,886
25-29	48	31
30-34	236	70
35-39	783	300
40-44	1,173	555
45-49	1,342	808
50-54	1,611	882
55-59	1,373	790
60-64	628	300
65-69	254	101
70-74	126	49

### COPD diagnosis

Spirometry (DISCOM 21 or MICROSPIRO HI-501; CHEST MI., Ins., Tokyo, Japan) was performed by experienced technicians. The largest value was chosen from among single and multiple spirometry examination results. FVC and FEV_1_ were measured, and FEV_1_/FVC was calculated. According to the standard criteria using the GOLD guidelines, subjects were diagnosed as COPD for FEV_1_/FVC less than 70%.

### Smoking status

A self-administered questionnaire on smoking status was used, including the number of cigarettes per day and the years since starting smoking. Subjects were classified by smoking status into three groups: non-smokers, former smokers and current smokers. Current smokers were classified by the Brinkman Index (BI) into three groups: BI <400, 400-799, and 800+. The BI was determined as the number of cigarettes per day multiplied by the years since starting smoking.^16^

### Data analysis

Data from the subjects mentioned above were available for sex, age, smoking status, BI, FVC and FEV_1_, but did not include any personal identifiers, such as name and address. To evaluate the association between age and COPD, logistic regression analyses with or without COPD as a dependent variable and age as an independent variable were conducted among both male and female non-smokers.

To evaluate the association between smoking and COPD adjusted for age, the ratio of the observed number of COPD cases to their expected number (O/E) was calculated for each group, i.e., former smokers, current smokers and current smokers of BI <400, 400-799, and 800+ among males and females. The expected number of COPD cases in each group was estimated as the total of the expected proportions of COPD among their subjects, which were calculated by the estimated logistic regression equation for non-smokers and their ages.

The statistical significance of O/E was tested under the assumption that the observed number of COPD cases assumed a Poisson distribution. The 95% confidence interval (CI) of O/E was estimated by an approximate method. If there were no observed COPD cases, an exact method was used. The trend in O/E over BI categories was tested by the Mantel test. The scores of BI categories of BI <400, 400-799, and 800+ were given as 200, 600 and 1,000, respectively. These analyses were performed using an SPSS^®^ 10.0J software package (SPSS Japan Inc.).

## RESULTS

### COPD and smoking status

Table 2 shows the number of COPD cases by smoking status. The number of current smokers was 3,564 (47.1%) in males and 203 (5.7%) in females, while the number of COPD cases was 188 (2.4%) in males and 25 (0.6%) in females. The proportion of COPD cases among current smokers was higher than that among non-smokers. The number of COPD cases in each BI group was more than 20 in males and less than 2 in females. The proportion of COPD cases increased with BI.

**Table 2.** Characteristics of subjects by sex and smoking status.

		Males		Females	

		n	cases of COPD* (%)	n	cases of COPD* (%)
Total		7,574	188(2.4)	3,886	25(0.6)
Never smokers		1,890	19(1.0)	3,565	20(0.6)
Former smokers		2,120	51(2.4)	118	0(0.0)
Current smokers		3,564	188(3.3)	203	5(2.5)
Brinkman Index^†^	<400	900	20(2.2)	161	2(1.2)
	400-799	1,685	43(2.6)	34	2(5.9)
	800+	979	55(5.6)	8	1(12.5)

* : Chronic obstructive pulmonary disease.

† : Brinkman Index: number of cigarettes per day × total number of years smoking.

### Association between COPD and age

**Figure 1.**
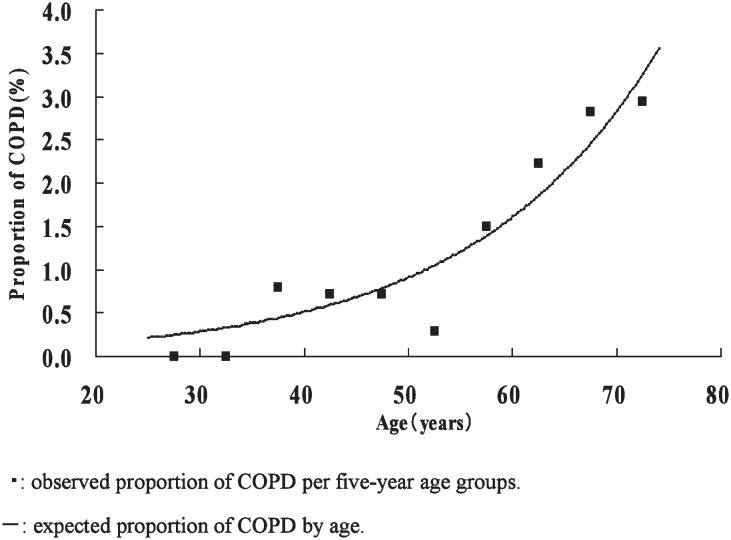
Proportion of chronic obstructive pulmonary disease cases by age in male non-smokers.

**Figure 2.**
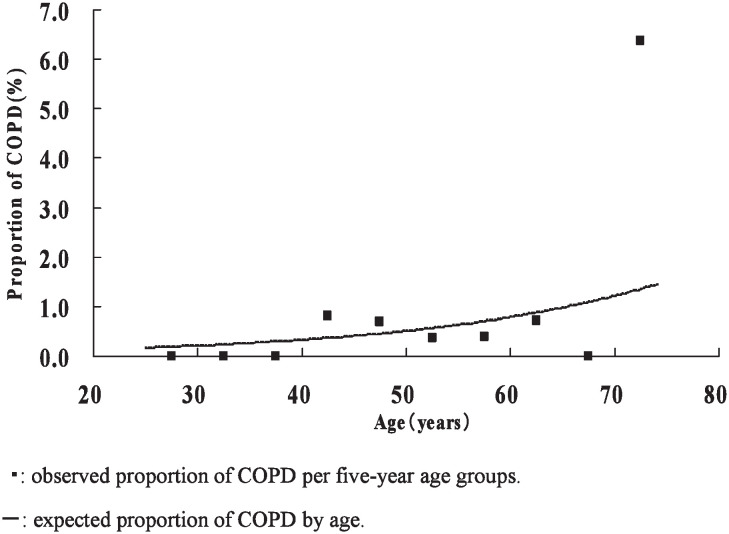
Proportion of chronic obstructive pulmonary disease cases by age in female non-smokers.

The curves of estimated proportions of COPD cases by age among non-smokers and those observed in the age groups are illustrated in Figure 1 for males and in Figure 2 for females. The proportion of COPD cases by age was estimated as 1/[1 + exp (7.595 - 0.058 × age)] in males and 1/[1 + exp (7.482 - 0.044 × age)] in females. Those estimates were comparatively consistent with the observed proportions in age groups except for the 70-74 year female age group. The estimated proportion significantly increased with age in males (p = 0.02), but not in females (p = 0.11). The increase per 10 years of age in the estimated proportion was 1.79-fold in males and 1.55-fold in females.

### Association between COPD and smoking status

**Figure 3.**
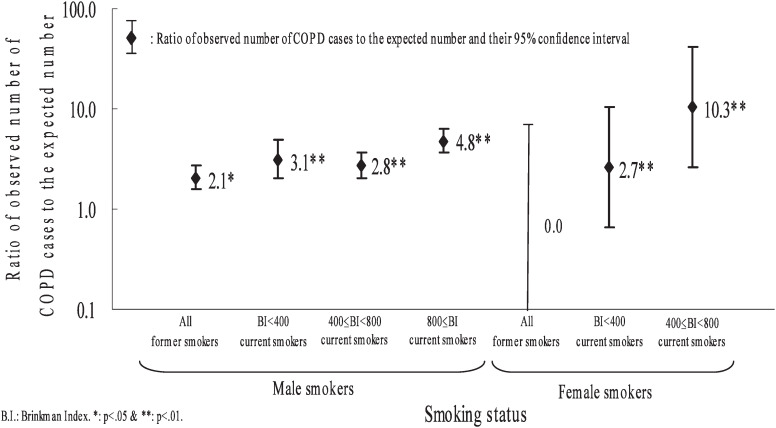
Ratio of observed number of chronic obstructive pulmonary disease cases to the expected number by smoking status.

Figure 3 shows the ratio of the observed number of COPD cases to the number expected for non-smokers with the same age distribution (O/E) by smoking status, excluding O/E in female current smokers with 800+ BI because of their small numbers. Among males, the O/E for former and current smokers was significantly higher than one. O/E for current smokers with BI <400, 400-799, and 800+ were 3.10 (95% confidence interval [CI], 2.00-4.81), 2.78 (2.05-3.73) and 4.76 (3.65-6.19), respectively. The trend in O/E over the BI categories was statistically significant (p = 0.03). Among females, the O/E for current smokers with BI <400 and 400-799 were significantly higher than one. The 95% CI of O/E for former and current smokers was extremely large.

## DISCUSSION

Many epidemiologic studies have reported that FEV_1_/FVC decreased with age,^17^^-^^19^ whereas there have been only a few studies on the association between COPD and age. The South Korea Study indicated that the proportion of cases with FEV_1_/FVC < 75% among males and females 45 years old or older was 4.3 times higher than among younger subjects.^8^ Our study confirmed that the proportion of COPD cases increased with age in male non-smokers in Japan under the standard diagnosis criteria for COPD. We failed to detect any significant association between COPD and age in female non-smokers. This might be due to the fact that our subjects were younger than 75 years of age, did not include many older females, or either that collecting accurate information about smoking was difficult in females, or that the increase with age in COPD cases among female non-smokers was lower than among male non-smokers.

Our study showed that COPD was significantly associated with smoking status in both males and females in Japan. The South Korea Study indicated that the age-adjusted odds ratio of cases with FEV_1_/FVC < 75% in smokers with BI 400+ compared with non-smokers was 3.2.^8^ A Greek study reported that the age-adjusted odds ratio of COPD in smokers with BI ≥ 300 compared with smokers with BI <300 was 1.5 in males and 4.7 in females under the standard diagnostic criteria for COPD, but it did not report comparative results between smokers and non-smokers.^12^ In our study, O/E for current smokers with BI <400, 400-799, 800+ were 3.10, 2.78, and 4.76, respectively, suggesting a dose-response relationship between COPD and smoking for males in Japan. These findings indicated that smoking constituted a strong risk factor for COPD. O/E for former smokers in males was 2.06. Smoking years after former smokers quit and quantities per day would be given more consideration.

There are several limitations and problems in our study. Although the GOLD diagnostic criterion of COPD is a recognized standard, it is not perfect. It might be important for diagnosing COPD to examine measures other than FEV_1_/FVC such as some definitions for airway obstruction^20^ and symptoms or histories related to COPD.^15^ Because those with diseases such as asthma and tuberculosis would not be suitable subjects for the criteria due to the difficulty in accurately measuring FVC and FEV_1_ by spirometry, then were excluded from our analysis. Although spirometry was performed by experienced technicians in our study, the FVC and FEV_1_ would inevitably include measurement variations. Moreover, our subjects were participants in a health check-up at a community medical center rather than being randomly selected from a community population, and they did not include many female smokers because of the relatively low smoking rate among Japanese females. Our study design was cross-sectional, whereas a large-scale longitudinal study would be important for more accurately evaluating the effects of age and smoking on COPD.
